# Time Trends of the Public’s Attention Toward Suicide During the COVID-19 Pandemic: Retrospective, Longitudinal Time-Series Study

**DOI:** 10.2196/24694

**Published:** 2020-12-30

**Authors:** Dayle Burnett, Valsamma Eapen, Ping-I Lin

**Affiliations:** 1 Department of Women's and Children's Health Uppsala University Uppsala Sweden; 2 School of Psychiatry University of New South Wales Kensington Australia; 3 South Western Sydney Local Health District Liverpool Australia

**Keywords:** COVID-19, suicide, infodemiology, infoveillance, Google Trends, time trend, school closure, attention, mental health, crisis, time series

## Abstract

**Background:**

The COVID-19 pandemic has overwhelmed health care systems around the world. Emerging evidence has suggested that substantially few patients seek help for suicidality at clinical settings during the COVID-19 pandemic, which has elicited concerns of an imminent mental health crisis as the course of the pandemic continues to unfold. Clarifying the relationship between the public’s attention to knowledge about suicide and the public’s attention to knowledge about the COVID-19 pandemic may provide insight into developing prevention strategies for a putative surge of suicide in relation to the impact of the COVID-19 pandemic.

**Objective:**

The goal of this retrospective, longitudinal time-series study is to understand the relationship between temporal trends of interest for the search term “suicide” and those of COVID-19–related terms, such as “social distancing,” “school closure,” and “lockdown.”

**Methods:**

We used the Google Trends platform to collect data on daily interest levels for search terms related to suicide, several other mental health-related issues, and COVID-19 over the period between February 14, 2020 and May 13, 2020. A correlational analysis was performed to determine the association between the search term ‘‘suicide’’ and COVID-19–related search terms in 16 countries. The Mann-Kendall test was used to examine significant differences between interest levels for the search term “suicide” before and after school closure.

**Results:**

We found that interest levels for the search term “suicide” statistically significantly inversely correlated with interest levels for the search terms “COVID-19” or “coronavirus” in nearly all countries between February 14, 2020 and May 13, 2020. Additionally, search interest for the term ‘‘suicide’’ significantly and negatively correlated with that of many COVID-19–related search terms, and search interest varied between countries. The Mann-Kendall test was used to examine significant differences between search interest levels for the term “suicide” before and after school closure. The Netherlands (*P*=.19), New Zealand (*P*=.003), the United Kingdom (*P*=.006), and the United States (*P*=.049) showed significant negative trends in interest levels for suicide in the 2-week period preceding school closures. In contrast, interest levels for suicide had a significant positive trend in Canada (*P*<.001) and the United States (*P*=.002) after school closures.

**Conclusions:**

The public’s attention to suicide might inversely correlate with the public’s attention to COVID-19–related issues. Additionally, several anticontagion policies, such as school closure, might have led to a turning point for mental health crises, because the attention to suicidality increased after restrictions were implemented. Our results suggest that an increased risk of suicidal ideation may ensue due to the ongoing anticontagion policies. Timely intervention strategies for suicides should therefore be an integral part of efforts to flatten the epidemic curve.

## Introduction

In March 2020, the World Health Organization (WHO) declared the COVID-19 outbreak a pandemic. To curb the spread of the SARS-CoV-2 virus, governments worldwide have implemented public health measures, such as lockdown, isolation, and social distancing [1,2]. Although these measures are needed to protect physical health, the psychological impact of the pandemic is still emerging. To alleviate the concerns surrounding mental health, there has been a growing interest in telepsychiatry, which is defined as “the delivery of mental health care in the form of live and interactive videoconferencing’’ [3]. Telepsychiatry has proven to be an effective approach for helping people access mental health services in remote and underserved areas [4,5]. Although the implementation of such services may be useful during the pandemic, the full impact of COVID-19 is unlikely to be fully understood for some time.

Ongoing measures, such as isolation and social distancing, have separated people from their loved ones, leading to loneliness, boredom, and stress. Recent mental health studies on COVID-19 have revealed that these feelings are associated with common mental health disorders, such as depression and anxiety [6-8], and that the most vulnerable are those with preexisting mental health disorders [3-5]. Moreover, emerging evidence has suggested that the environmental nature of public health measures (eg, locations where quarantine is carried out) can lead to different effects on mental health. For instance, a recent study has revealed that poor housing is associated with an increased risk of depressive symptoms during lockdown [9].

Previous research on the psychological impact of quarantine from past epidemics has revealed that the fear of infection and lack of clear communication from governments are associated with a high prevalence of anxiety, exhaustion, psychological distress, and depression [10]. Furthermore, it has been reported that the lockdown, which has been enforced in many countries around the world, will have a substantial impact on the global economy; there have been predictions of an economic crisis and a large increase in unemployment worldwide, and unemployment is a well-recognized risk factor for suicide [11].

There is a growing body of literature that suggests suicide rates will increase during and after the pandemic due to anticontagion policies that increase social isolation and loneliness, which are well-known risk factors for suicide [12-15]. Indeed, there have been cases of COVID-19–related suicides in the United States, United Kingdom, Italy, Germany, Bangladesh, and India [14]. There have also been suggestions that the suicide rate will rise as the full economic and social impact of the pandemic unfolds. For example, higher suicide rates were observed among older adults in Hong Kong during the 2003 severe respiratory syndrome outbreak [16,17].

A considerable amount of published literature has examined the effects of the COVID-19 pandemic on the mental health of the general population; vulnerable people, including older adults and patients with chronic health conditions; patients with previous mental health disorders; and health care professionals [14,18-24]. These studies have indicated that suicide is likely to become an increasing issue as the pandemic continues, and that the pandemic has a long-term effect on the general population, not just those who are at an increased risk of suicide. Thus, to reduce the incidence of suicide during the COVID-19 pandemic, it essential to mitigate risk factors, such as stress, anxiety, fear, and loneliness, in the general population.

Although reducing the number of social interactions remains one of the best methods to reduce the total burden of COVID-19, questions have been raised about control measures, such as school closure. The effectiveness of school closures has been suggested in previous studies on influenza outbreaks, which have reported that influenza transmission is higher in children than in adults [25]. However, in the context of COVID-19, data have indicated that COVID-19 transmission dynamics are different from influenza transmission dynamics; adults and older adults are more vulnerable to COVID-19 than the general population [26]. Furthermore, school closures can have large implications on society. In a recent review of school closure practices, Viner et al [27] found that such measures alone would only prevent 2%-4% of deaths, which is a much lower death prevention rate than that of other social distancing interventions. Viner et al [27] noted that the negative consequences of school closures include economic costs due to parents missing work to look after their children and reductions in health care staff resources, which in turn can negatively impact health care systems.

Due to the fast-moving nature of the pandemic, real-time data collection is needed to assess public interest in COVID-19. To date, the internet has been increasingly used as a source of health care information, especially Google, which is the world’s most used search engine [28]. Google Trends is a website created by Google that analyses the popularity of the top search queries in Google Search; Google Trends has proven to be a powerful tool in tracking public interest in infectious diseases [29]. This study aims to explore the trends of COVID-19–related search terms and their association with common mental health disorders. In addition, this study aims to determine whether school closure, which we used as a proxy for nonpharmaceutical anticontagion policies, is associated with the risk of suicide.

## Methods

### Data Source

We used the web tool Google Trends to quantify web-based search interest in this study. The methodology we designed was based on the Google Trends Methodology Framework in Infodemiology and Infoveillance [30]. Google Trends does not show actual search volume numbers. Instead, Google Trends provides the number of relative searches within a specified region and time for a particular search query by using a scale of 0-100. A value of 100 indicates the peak popularity of the query, whereas a score of 0 indicates a very small number of searches.

Data from Google Trends was compiled between February 14, 2020 and May 13, 2020. The following 16 countries were assessed in this study: Australia, Austria, Belgium, Canada, France, Germany, Ireland, Italy, the Netherlands, New Zealand, Portugal, Russia, Spain, Sweden, the United Kingdom, and the United States. The countries were chosen to represent locations in Europe with the largest number of COVID-19 deaths or those that were forecasted to experience a considerable number of deaths. We also included English-speaking countries outside of Europe, as our study was conducted in English. To determine whether the time since school closures correlated with the stringency level of anticontagion policies, we extracted stringency index data from the Our World in Data database [31] and school closure dates from the datasets provided by the United Nations Education, Scientific and Cultural Organization [32].

### Search Terms

We used the following search queries to examine common mental health disorders: “suicide,” “depression,” and “anxiety.” To investigate any potential confounding factors related to suicide, we performed a manual search of the term ‘‘suicide’’ to find data on the suspected suicides of celebrities in the countries used in this study, by using advanced Google searches and web-based news articles. We tailored the dates to include results from between February 14 and May 13, 2020. We also studied the following COVID-19–related search queries: “Coronavirus (Virus),” “social distancing,” “school closure,” “self-isolation,” and “lockdown.” The search queries were chosen based on a recent review on suicide risk and prevention during the COVID-19 pandemic, in addition to keywords that were used in the media and government and WHO policy briefings [12]. It should be noted however that the term “Coronavirus (Virus)” was searched as a topic, which is defined as a group of terms that share the same concept in any language. This was done to include data on the different names associated with coronavirus, such as COVID-19. Although Google Trends provides users with the opportunity to compare up to 5 search queries at the same time, we decided to extract data for each search query individually and compare each query in its own distribution. The search was carried out using English terms.

### Data analysis

#### Primary Analysis

We investigated the changes in trends for all the different search queries in Google Trends by using graphical representations for each country. This was carried out by using the smooth splines function in the ggplot2 package of R Studio. We used the Pearson correlation test to measure the strength of the association between each search query for each country. A 2-sided α value of <.05 was used as a cutoff to identify countries that showed a significant association between the terms ‘‘school closure’’ and ‘‘suicide’’ for the next part of the analysis. The relationship between the time to school closure and the stringency index was examined using a generalized linear model, and the generalized estimating equation was used to correct for intracountry correlations [33].

#### Secondary Analysis

We carried out a Mann-Kendall test to compare the trends in suicide interest before and after school closures. We first identified the national school closure date for each of the selected countries, and then extracted Google Trends data for the search term ‘‘suicide’’ at 2 weeks before and after the school closure date for each country. The Mann-Kendall method is a nonparametric test that is used to detect statistically significant trends [34,35]. In this test, the null hypothesis (H_0_) was that there was no trend in the number of suicide searches over time. The alternative hypothesis (H_1_) was that there was a trend over time.

The Mann-Kendall *S* statistic was calculated using the following formula:


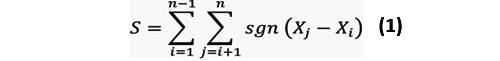


In this formula, x_j_ and x_i_ are time series and n is the number of data points in the time series. With regard to calculating sgn, the following formula was used:


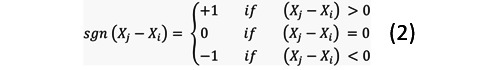


The variance of the Mann-Kendall test was calculated as follows:


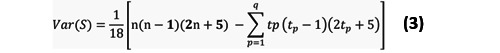


In this formula, q is the number of tied groups and t_i_ is the number of data values in the pth group.

The standard test statistic *Z* was calculated as follows:


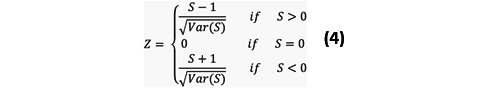


*Z* follows the standard normal distribution. A positive *Z* value indicates a positive trend, whereas a negative *Z* value indicates a negative trend.

## Results

### Graphical Analysis

**Figure 1:**
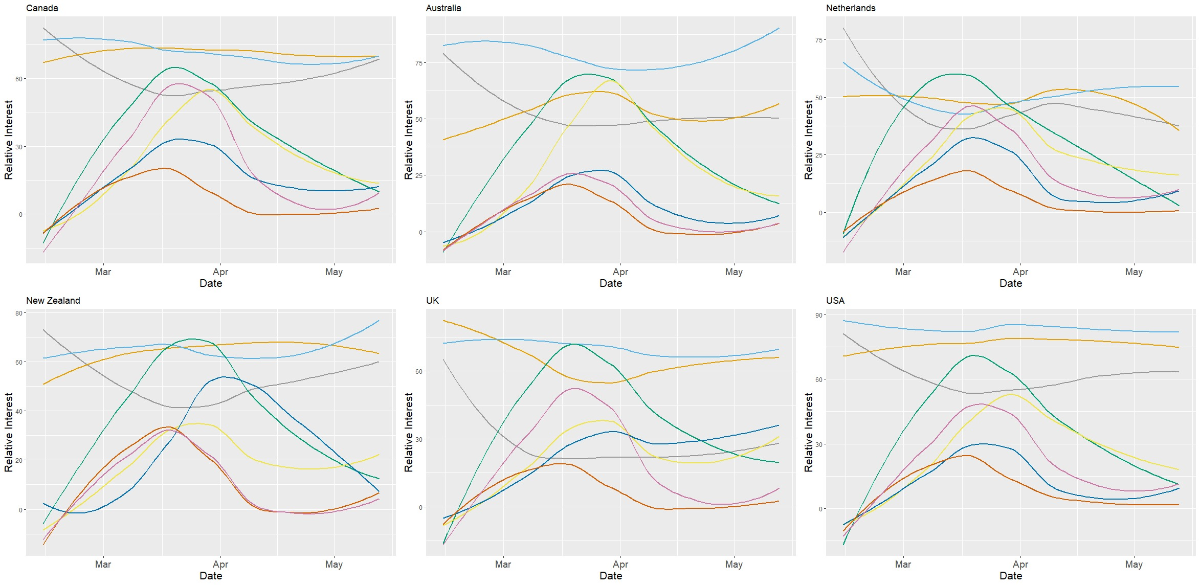
Google Trends data for the selected 6 countries from between February 14, 2020 and May 13, 2020. The grey curve represents the relative interest for the term ‘‘suicide,’’ the orange curve represents the relative interest for the term ‘‘depression,’’ the light blue curve represents the relative interest for the term ‘‘anxiety,’’ the green curve represents the relative interest for the term “Coronavirus (Virus),” the yellow curve represents the relative interest for the term “social distancing,” the dark blue curve represents the relative interest for the term ‘‘lockdown,’’ the red curve represents the relative interest for the term “school closure,” and the purple curve represents the relative interest for the term ‘‘self-isolation." UK: United Kingdom; USA: United States of America.

We assessed Google Trends data from the period between February 14, 2020 and May 13, 2020 for the selected search queries in the selected countries, as displayed in Figure 1. What stands out in all the figures are the common trends in COVID-19–related search queries across several countries. With regard to the search query ‘‘Coronavirus (Virus),’’ most countries showed a noticeable increase in the number of searches for terms related to coronavirus from the end of February until the middle of March, which is when interest for the term peaked, followed by a decline in the number of searches. The peak in interest was most likely due to the WHO declaring COVID-19 a pandemic.

Although lockdown, social distancing, and self-isolation have been the main strategies for reducing the spread of COVID-19, it is clear from the graphs in the figures that the public’s level of interest varied between countries and the different measures taken to combat the virus. For example, there was a greater interest in searches for the term “lockdown” in Germany, New Zealand, Portugal, and Russia compared to other countries with different public health measures. Surprisingly, there was a large number of searches in Sweden for the term “lockdown,” despite the fact that the government decided against imposing a lockdown, and instead relied on voluntary cooperation from its citizens [36].

In Australia, Austria, and Belgium, there was a greater interest in social distancing measures, while in the United Kingdom and Ireland, self-isolation generated greater interest. However, in Canada, the Netherlands, Spain, and the United States, the interest levels for self-isolation and social distancing produced very similar relative search volumes.

A closer inspection of the graphs revealed that the number of searches for the term “lockdown” in Austria, France, Ireland, Russia, Spain, and Sweden peaked at around the end of March, followed by a plateau. In other countries, the number of searches for “lockdown” decreased over time. In Germany, Portugal, and the United Kingdom, the interest levels for lockdown increased throughout the study period. Interestingly, searches for ‘‘lockdown’’ in Italy peaked during the middle of April. This could be due to Italy starting to ease lockdown measures, due to public interest in what activities could be carried out.

The interest levels for the term ‘‘school closures’’ varied between countries, and most countries showed a bell-shaped trend for the interest level. The variation in interest levels is most likely due to different countries announcing and enforcing school closures on different dates, as well as several countries imposing regional lockdowns before a national lockdown.

In most of the countries, the number of searches for the term “suicide” began to decrease toward the end of February and at the beginning of March. Afterward, the number of searches started to increase again in several countries, namely France, Spain, Russia, New Zealand, and the Netherlands. In other countries, such as Australia, Belgium, and the United Kingdom, the search levels for the term “suicide” remained constant. It should be noted however that the peak in suicide-related search terms during February in the United Kingdom and, to a lesser extent, Ireland might have corresponded to the suicide death of television presenter Caroline Flack on February 15, 2020. This possibility was also highlighted in a similar study [37].

Levels of anxiety varied between the countries. In Ireland, the United Kingdom, and the United States, search interest levels for the term “anxiety” remained high throughout the study period. In Australia, Germany, New Zealand, and Portugal, the interest levels for “anxiety” increased after public health measures were implemented.

### Correlational Analysis

**Figure 2:**
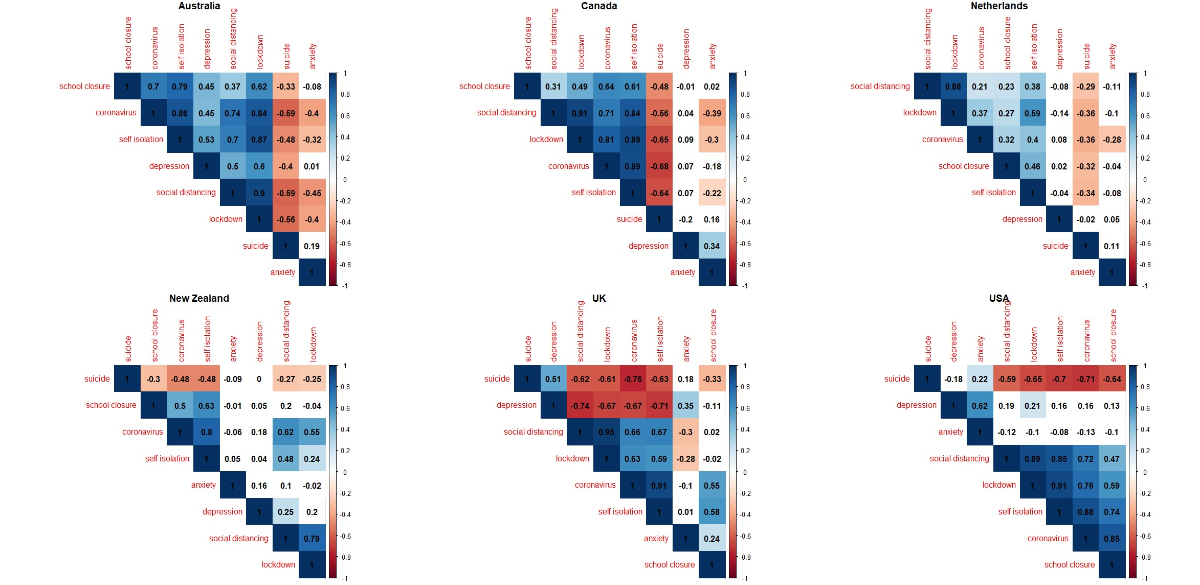
Correlation matrices that represent the pairwise Pearson correlation coefficients of Google Trends search queries for 6 selected countries. Colored cells represent a statistical significance level of *P*<.05. UK: United Kingdom; USA: United States of America.

The results of the correlational analysis for selected countries are shown in Figure 2. The most interesting aspect of this figure is that searches for the term ‘‘suicide’’ did not significantly positively correlate with COVID-19–related queries. In Australia, Canada, Ireland, New Zealand, the United Kingdom, and the United States (ie, all the English-speaking countries), as well as in the Netherlands, all COVID-19–related search queries were negatively associated with the term ‘‘suicide.’’ In Belgium, France, Italy, Spain, and Russia, searches for the term ‘‘suicide’’ were only significantly negatively associated with some of the other COVID-19-related queries.

The association between searches for the term ‘‘suicide’’ and searches for other mental health disorder–related terms varied between countries. Searches for the term ‘‘suicide’’ significantly positively correlated with searches for the term ‘‘depression’’ in France (*r*= 0.5401, *P*<.001), Germany (*r*=0.2976, *P*=.004), Russia (*r*= 0.3175, *P*=.002), and the United Kingdom (*r*= 0.5137, *P*<.001), which is to be expected, as depression is a well-known risk factor for suicide [12]. However, in Australia, a negative correlation was observed (*r*=−0.403, *P*<.001). In addition, there was a weak significant correlation between the interest levels for the terms ‘‘suicide’’ and ‘‘anxiety’’ in the United States. Significant associations were observed between the terms ‘‘anxiety’’ and ‘‘depression’’ in Canada (*r*=0.3429, *P*<.001), Russia (*r*=0.2389, *P*=.02), the United Kingdom (*r*=0.3506, *P*<.001), and the United States (*r*=0.6189, *P*<.001).

In most countries, strong positive correlations were observed between the search term ‘‘Coronavirus (Virus)’’ and other COVID-19–related search terms, which suggests that the public has been continuing to seek information about the SARS-CoV-2 virus and the measures that have been taken to curb the spread of the virus. The results obtained from the correlational analysis between the terms ‘‘school closure’’ and ‘‘suicide’’ are shown in Table 1; only countries that showed a significant relationship between interest levels are shown.

**Table 1:** Results of the Pearson coefficient test between interest levels for the terms ‘‘school closure’’ and ‘‘suicide’’ based on Google Trends data from countries that showed a significant relationship between interest levels.

Country	*r*	*P* value
Australia	−0.33	<.001
Canada	−0.48	<.001
Netherlands	−0.32	.002
New Zealand	−0.3	.003
United Kingdom	−0.33	.001
United States	−0.64	<.001

### Mann-Kendall Analysis

**Figure 3:**
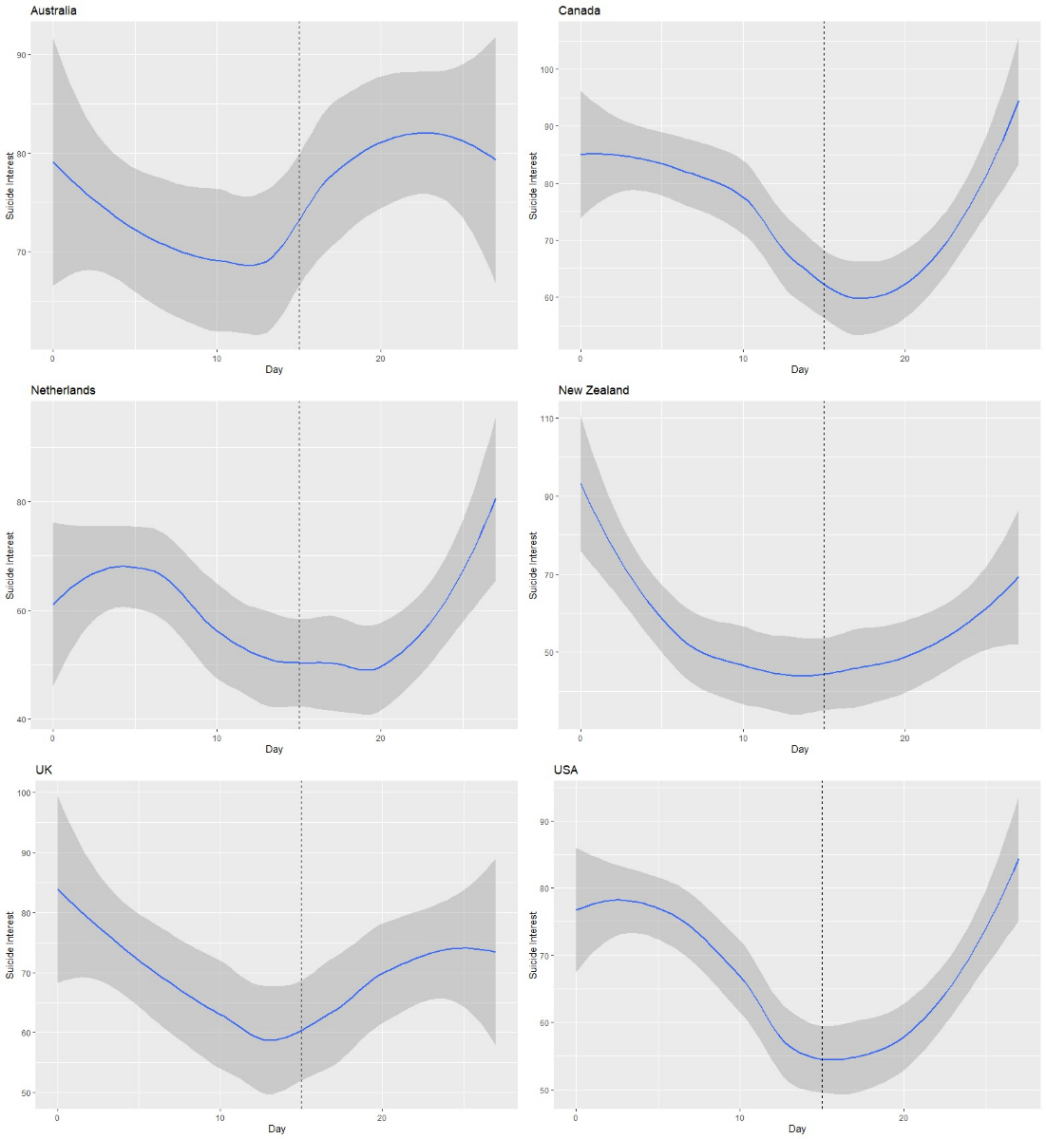
Relative search interest level for the term ‘‘suicide’’ during the school closure period in selected countries. The dashed vertical line indicates the national school closure date. UK: United Kingdom; USA: United States of America.

The time to school closure positively correlated with the stringency index level (coefficient: 0.97, *P*<.001). Figure 3 shows the trend in suicide-related search queries during the school closure period in countries where a significant association between the search terms ‘‘suicide’’ and ‘‘school closure’’ was found, based on Google Trends data. From the graphs, we can see that there was a decrease in the interest of suicide in all the countries before school closure, whereas an increase in the interest levels for suicide occurred after school closure.

The Mann-Kendall test was applied on a daily basis to detect trends in the interest levels for suicide before and after countries’ respective school closure period. Table 2 shows the test results in terms of the Mann-Kendall score (*S*) and *Z* statistic. Of the 6 countries, the Netherlands (*P*=.19), New Zealand (*P*=.003), the United Kingdom (*P*=.006), and the United States *(P*=.049) showed a significant negative trend in the interest levels for suicide in the 2-week period leading up school closure. New Zealand showed the largest decrease in the number of searches related to suicide in the 2 weeks leading up to the school closure. No significant trends were found in other countries. In contrast, the interest levels for suicide significantly positively correlated in Canada (*P*<.001) and the United States *(P*=.002) after schools closed. This trend was larger in Canada than in the United States.

**Table 2:** Mann-Kendall test results for the comparison between suicide interest levels 2 weeks before school closure and 2 weeks after school closure for selected countries.

Country	Before school closure			After school closure		
	*S*	*Z*	*P* value	*S*	*Z*	*P* value
Australia	−25	−1.31	.19	13	0.66	.51
Canada	−23	−1.21	.23	72	3.9	<.001
Netherlands	−25	−1.32	.19	34	1.81	.07
New Zealand	−54	−2.91	.003	29	1.53	.13
United Kingdom	−51	−2.75	.006	17	0.88	.38
United States	−36	−1.97	.049	57	3.07	.002

## Discussion

### Principal Findings

By using web-based search interest as a proxy for the public’s attention to specific terms, we found that the attention to terms related to COVID-19 surged following the COVID-19 outbreak in February 2020 and peaked between late March and mid-April 2020, which is when attention to suicide and other mental health issues started and continued to decline. Attention seems to have shifted from COVID-19 to suicide and other mental health-related issues after anticontagion policies, such as school closures, were enacted in many countries.

Our initial results are in line with recent studies, which have indicated that the number of searches related to COVID-19 rose during the peak of the pandemic [38-40]. However, we also found variations between different COVID-19–related search terms between different countries. This is most likely due to different countries being at different stages during the pandemic. Italy was the first country in Europe to implement a lockdown, and this was swiftly followed by many Western European countries implementing lockdown. Moreover, it is important to take into consideration the different strategies that were executed by different countries when interpreting these results. For example, while most countries in Europe implemented a full or partial lockdown, Sweden took a different route and emphasized the importance of social distancing.

In this study, we found that searches for the term “suicide” increased after anticontagion measures, such as school closures, were enforced. These findings are consistent with those from studies that used Google Trends to explore the changes in the public’s search behaviors during the pandemic [37,41]. A note of caution is due, however, when comparing this study to previous research, as our study used school closure during the pandemic as a proxy, whereas most studies have focused on lockdown. Nonetheless, school closures can be regarded as a form of quarantine, and the adverse effects of school closures mainly impact certain population groups, such as children and adolescents, parents, and health care workers [26].

### Limitations

Despite the strengths of this study, there are several limitations to consider when interpreting the results. First, this study focused on the 90-day period ensuing the outbreak. The results might have led to different conclusions if data from a longer period of time were analyzed. However, we intended to evaluate the short-term impact of anticontagion policies by using school closure as a proxy. Therefore, the selected period of time might provide more relevant information. Second, the data was extracted from Google Trends, which only provides data on relative search volumes. If absolute numbers were provided instead of relative search volumes, a better comparison between countries could have been made, and more accurate results could have been obtained. Third, we did not adjust for the possible confounding effects of sociodemographic factors in the analyses. Economic downturn factors, such as job loss, may mediate or confound the association between the COVID-19-related events and the public’s interest in suicidality. However, the goal of this study was to identify patterns in the public’s attention toward suicidality in relation to COVID-19 instead of focusing on the causal relationship between the two. Therefore, the lack of taking mediators or confounders into consideration would not have affected our conclusions. However, the validity of Google Trends search interest for the behavioral forecasting of suicide rates may be low, because users’ characteristics and motivations are unknown [42]. A recent study has shown that web-based search interest levels with regard to suicidality may concurrently correlate with the volume of aggregate incidents of suicidality at the population level [43]. Therefore, we believe that this study can provide insight into the temporal trend of imminent suicidal risk in different populations. Fourth, only English search terms were used in this study, and this could potentially restrict one’s ability to make generalizations based on the study findings. We chose not to translate search terms because this approach requires a comprehensive understanding of each language to ensure that the key concepts of search terms are studied sufficiently. Although Google Trends provides users with the option of using topics (ie, a group of search terms that Google identified to share similar meanings across languages and countries), search terms such as “school closure” and “self-isolation” were unavailable at the time of writing. This approach could lead to obtaining data from users that only searched for a term in English in non-English-speaking countries (eg, France). Further, we examined the search interest levels for the term “lockdown” in France as an example, based on separate Google “Topics” and “Search Term” values. We found that these 2 values highly correlated with each other (*r*=0.95, *P*<.001) in countries where English is not the primary language, such as France. Since our study did not intend to compare search interest levels between different countries, this limitation does not affect our conclusion. Finally, this study only used Google Trends to compare time trends during the pandemic. However, we do recognize that other platforms, such as Twitter, may have also been useful in identifying the public’s concerns.

### Public and Clinical Implications

Based on the growing body of literature on the impact of COVID-19, it is clear that mental health and psychological considerations are paramount. The findings from this study validate and extend previous work that used Google Trends to track public concerns.

The use of Google Trends in this study has important policy implications. For example, school closures have led to an increase in searches for the term “suicide,” which suggests that people’s mental health may have been affected by school closures. Thus, policymakers will need to face the challenges of combating the virus through anticontagion policies while also reducing unintended consequences, particularly the consequences on mental health.

To conclude, our findings suggest that the consequences of the COVID-19 pandemic vary depending on countries’ public health anticontagion measures. Although our study focused on high-income countries, it is important to recognize the potential impact our findings have on resource-poor settings, where mental health support is lacking. We believe that our results provide insight into the importance of integrating mental health services into anticontagion policies, as there is an urgent need to address how the mental health consequences of the pandemic can be mitigated, should similar outbreaks occur in the future [44].

## Abbreviations

WHO: World Health Organization
